# *OsFON879* , an orphan gene, regulates floral organ homeostasis in rice

**DOI:** 10.1111/pbi.70121

**Published:** 2025-05-03

**Authors:** Dong‐Hui Wang, Zhi‐Hong Xu, Shu‐Nong Bai

**Affiliations:** ^1^ State Key Laboratory of Protein and Plant Gene Research, School of Life Sciences Peking University Beijing China

**Keywords:** *Oryza sativa*, orphan gene, floral organ homeostasis, RNA‐binding protein, *OsFON879*, *OsRRM1*

Floral organ development in angiosperms is tightly regulated by conserved pathways, including the ABCE model and *CLV‐WUS* signalling, which coordinate meristem activity and organ identity (Litt and Kramer, 2010; Schoof *et al*., 2000). However, the mechanisms underlying species‐specific floral architecture, particularly in monocots like rice (Oryza sativa), remain poorly understood. Although key regulators such as *FON1/FON2* and ABCE‐class genes coordinate floral meristem maintenance (Hu *et al*., 2015; Ren *et al*., 2019; Suzaki *et al*., 2004), the contribution of lineage‐specific genetic innovations, particularly orphan genes (OGs), to floral organ development remains poorly characterized.

OGs, which lack detectable homologues or conserved domains across species, have emerged as key contributors to species‐specific traits and adaptive evolution (Tautz and Domazet‐Lošo, 2011). Despite their importance, OGs remain understudied due to challenges in functional characterization. Recent studies have implicated OGs in specialized developmental processes (Jiang *et al*., 2022), but their precise roles in plant development are not well understood. Here, we characterize *OsFON879* (*LOC_Os03g12879*), a rice‐specific OG encoding a predicted intrinsically disordered protein (IDPs) based on AlphaFold (Figures S1–S3). IDPs are known to act as dynamic scaffolds, facilitating interactions between multiple macromolecules (e.g., proteins, RNA) in regulatory hubs (Chakrabarti and Chakravarty, 2022). *In situ* hybridization (Figure 1a1–a6) and RT‐qPCR assay (Figure 1a7) revealed *OsFON879* expression specifically in reproductive tissues, peaking during early panicle development. These results indicate that the IDP of *OsFON879* may play an important role in the floral development process of rice.

**Figure 1:**
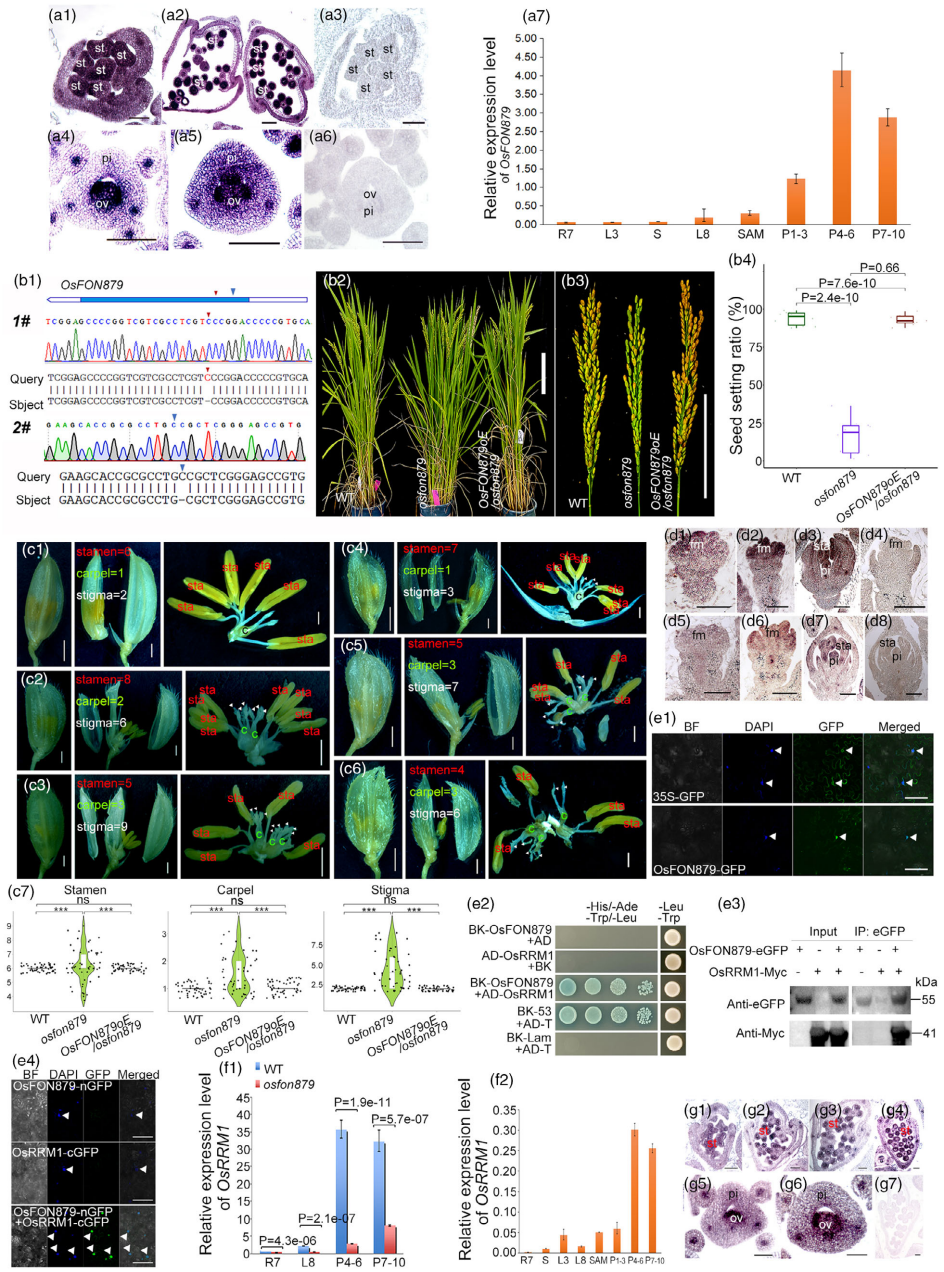
*OsFON879* regulates floral organ development in rice. (a1–a6) *In situ* hybridization of *OsFON879* in rice stamens (a1–a2) and carpels (a4–a5); sense strand controls (a3, a6) show no signal. st, stamen; pi, pistil; ov, ovary. Scale bars: 50 μm. (a7) RT‐qPCR analysis of *OsFON879* expression in root meristems (R7), mature leaves (L3, L8), seedlings (S) and panicles at developmental stages P1‐3 (1–3 mm), P4‐6 (4–6 mm) and P7‐10 (7–10 mm). Data normalized to *GAPDH*; mean ± SD (*n* = 3). (b1–b4) Comparison of wild‐type (WT) and *osfon879* mutant plants. Arrow indicates CRISPR‐Cas9 mutation site. The mutant alleles (*osfon879‐1#* and *osfon879‐2#*) were generated using two distinct sgRNAs targeting exon regions, resulting in frameshift mutations. The target sequences are in Table S1. Seed fertility is reduced in mutants (b3‐b4) but restored in complementation lines (b2‐4). Phenotypic differences analysed by Student's *t*‐test. Scale bar: 20 cm (1b2); 10 cm (1b3). (c1–c6) Floral organ phenotypes in WT (c1) and *osfon879* mutants (c2–c6). The white triangle indicates the stigma. sta, stamen; c, carpel. Scale bar: 1 mm. (c7) Statistical distribution of floral organ numbers in WT, *osfon879* mutants and complementation lines (*OsFON879oE/osfon879*) by Violin plots with box plots (*n* = 50 per group; ***p < 0.001, *T*‐test). (d1–d8) In situ hybridization of *OsMADS1* in WT (d1‐d3) and *osfon879* mutant (d5–d7) flowers. Sense controls (d4, d8) show no signal. fm, floral meristem; pi, pistil; sta, stamen. Scale bars: 100 μm. (e1) Subcellular localization of OsFON879 in *Nicotiana benthamiana* epidermal cells. Top, localization of signal from empty vector containing GFP alone. Bottom, localization of OsFON879 fused with GFP. Scale bar: 200 μm. (e2–e4) OsFON879 interacts with OsRRM1: yeast two‐hybrid assay (e2), co‐immunoprecipitation (Co‐IP, e3) in protoplasts of rice and bimolecular fluorescence complementation (BiFC, e4) in tobacco. BK‐53 + AD‐T serves as the positive control, and BK‐Lam + AD‐T serves as the negative control. Scale bar: 200 μm. (f1) *OsRRM1* expression is reduced in *osfon879* mutants by RT‐qPCR assay. RT‐qPCR data from R7, L8, P4‐6 and P7‐10 according as above (mean ± SD, *n* = 3; Student's *t*‐test). (f2) *OsRRM1* expression pattern in WT by RT‐qPCR assay. RT‐qPCR data from R7, S, L3, L8, P1‐3, P4‐6 and P7‐10 according to above (mean ± SD, *n* = 3; Student's *t*‐test). (g1– g7) *In situ* hybridization of *OsRRM1* in stamens (g1–g4) and carpels (g5–g6); sense control (g7) shows no signal. Scale bars: 50 μm. Specific primers for each target gene are listed in Table S2.

CRISPR‐Cas9‐induced mutant *osfon879* exhibited stochastic floral organ defects and a moderate increase in tiller number during panicle development, which were fully rescued in genetic complementation lines (Figure 1b1–b4). Twenty‐four per cent of the *osfon879* mutant flowers had fewer stamens (minimum of four per flower), and 30% had more (up to nine per flower). Also, 70% of mutant flowers had more stigma (up to nine per flower). The carpels were affected too, with 32% of flowers having two and 12% having three. A few spikelets had more glumes (Figure 1c1–c7). The stochastic floral organ patterning in *osfon879* mutants implicates a role in maintaining meristem homeostasis; however, its lack of conserved protein domains implies a novel regulatory mechanism diverging from canonical pathways.

*In situ* hybridization demonstrated suppressed *OsMADS1* expression in *osfon879* mutants (Figure 1d1–d8). Given that OsMADS1, an E‐class MADS‐box gene, is critical for floral organ identity specification (Je *et al*., 2016), this implies *OsFON87*9 modulates *OsMADS1* expression. The subcellular localization results indicate that *OsFON879* is predominantly localized in the nucleus, with smaller amounts present in the cytoplasm and cell membrane (Figure 1e1). Yeast two‐hybrid screening revealed that OsRRM1, an RNA‐binding protein, interacts with OsFON879 (Figure 1e2). The OsFON879‐OsRRM1 interaction was independently confirmed through co‐immunoprecipitation (Co‐IP) in rice 7‐day root protoplast systems (Figure 1e3) and bimolecular fluorescence complementation (BiFC) assays in *Nicotiana benthamiana* epidermal cells (Figure 1e4), definitively confirming their direct physical association. In *osfon879* mutants, the expression of *OsRRM1* was significantly decreased (Figure 1f1). Furthermore, RT‐qPCR (Figure 1f2) and *in situ* hybridization analyses (Figure 1g1–g7) revealed a spatiotemporal co‐expression pattern of *OsRRM1* and *OsFON879*, with both genes exhibiting high and tissue‐specific expression in rice floral organs. These findings reveal that *OsFON879* directly regulates the expression of *OsRRM1*, highlighting their functional interdependence.OsRRM1 contains an RNA recognition motif (RRM) which is predicted to bind mRNAs encoding floral meristem regulators (e.g., *OsMADS1* and *OsWUS*) based on RiceFREND database analysis (Sato *et al*., 2013). The interaction and spatiotemporal coexpression of *OsFON879* and *OsRRM1* in floral organs, combined with reduced *OsRRM1* expression in *osfon879* mutants, supports a model where *OsFON879* stabilizes OsRRM1 activity. We propose that *OsFON879* recruits OsRRM1 to modulate RNA stability or translation efficiency of target transcripts, thereby regulating meristem activity. This hypothesis is consistent with the stochastic floral organ phenotypes observed in *osfon879* mutants, which reflect disrupted RNA processing. Importantly, the regulatory effect of *OsFON879* on *OsMADS1* expression level in the *osfon879* mutant identifies OsFON879 as a central player in floral organogenesis. We propose that the OsFON879‐OsRRM1 interaction stabilizes OsMADS1 transcripts, ensuring robust E‐class function during floral organ specification.

This study identifies *OsFON879* as a rice‐specific OG critical for floral organ development regulation. *OsFON879*'s orphan status and lack of conserved domains suggest a novel regulatory layer. The interaction with OsRRM1 links orphan genes to RNA metabolism, expanding known floral meristem networks beyond transcriptional control. Our findings highlight orphan genes as untapped resources for engineering agronomic traits and advance understanding of post‐transcriptional regulation in plant development. Future studies should explore *OsFON879‐OsRRM1*'s role in transcriptome‐wide RNA processing and their genetic interactions with known meristem regulators.

## Funding

National Natural Science Foundation of China (31630006).

## Conflicts of interest

The authors declare no competing interests.

## Author contributions

D.H.W. designed and performed experiments and wrote the manuscript. Z.H.X. and S.N.B. supervised and revised the manuscript.

## Supporting information


**Figure S1** AlphaFold structural prediction of OsFON879. Highlighted regions: Low‐confidence/disordered segments (yellow) and very low‐confidence segments (orange) (https://alphafoldserver.com/).
**Figure S2** Showing features for region of OsFON879 with UniProt feature annotations. Highlighted regions: Disordered segments (orange) (https://www.uniprot.org/uniprotkb/Q10PL1/entry).
**Figure S3** Sequences producing significant alignments with NCBI BLAST (https://blast.ncbi.nlm.nih.gov/Blast.cgi).
**Table S1**. Target sequences used for CRISPR/Cas9‐mediated gene knockout.
**Table S2**. Primer pairs used in this study.

**Data S2.** Materials and Methods.

## Data Availability

The data that support the findings of this study are available on request from the corresponding author. The data are not publicly available due to privacy or ethical restrictions.
