# Supplementary material for: OsFON879 , an orphan gene, regulates floral organ homeostasis in rice
Source: Plant Biotechnol J. 2025 May 3;23(7):2888–90. doi: 10.1111/pbi.70121 (PMC12205862; doi:10.1111/pbi.70121)
Supplement: Supplementary file 1 — Figure S1 AlphaFold structural prediction of OsFON879. Highlighted regions: Low‐confidence/disordered segments (yellow) and very low‐confidence segments (orange) (https://alphafoldserver.com/). Figure S2 Showing features for region of OsFON879 with UniProt feature annotations. Highlighted regions: Disordered segments (orange) (https://www.uniprot.org/uniprotkb/Q10PL1/entry). Figure S3 Sequences producing significant alignments with NCBI BLAST (https://blast.ncbi.nlm.nih.gov/Blast.cgi). Table S1. Target sequences used for CRISPR/Cas9‐mediated gene knockout. Table S2. Primer pairs used in this study. [file PBI-23-2888-s001.docx]

**Supplemental Figure and Table**

**Supplemental Figure**


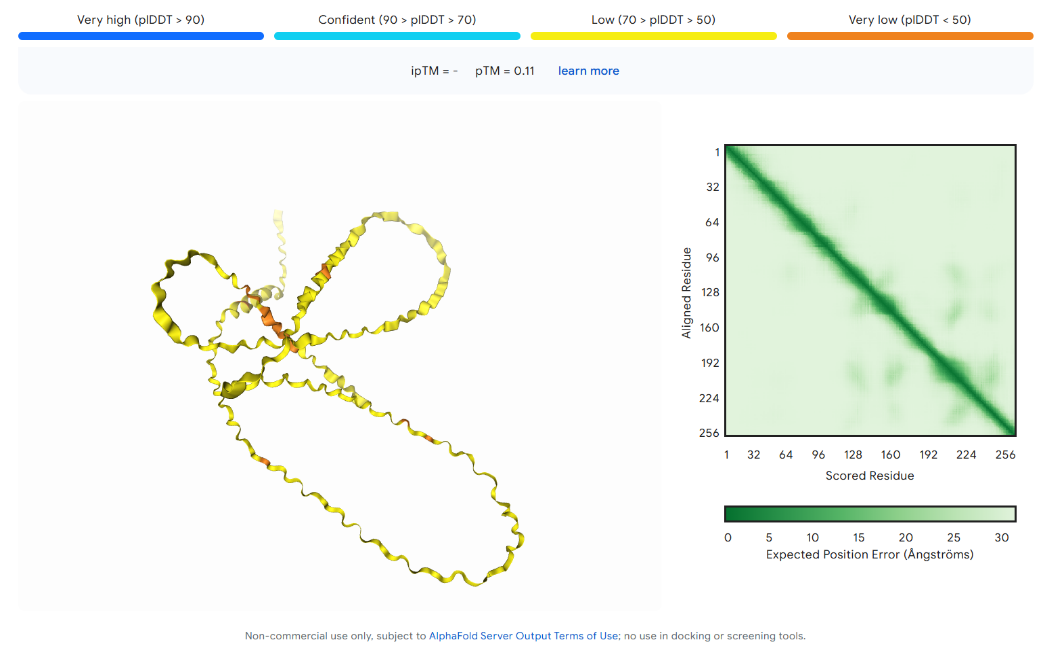


**Supplemental Figure S1** AlphaFold structural prediction of OsFON879. Highlighted regions: Low-confidence/disordered segments (yellow) and very low-confidence segments (orange) (<https://alphafoldserver.com/>).


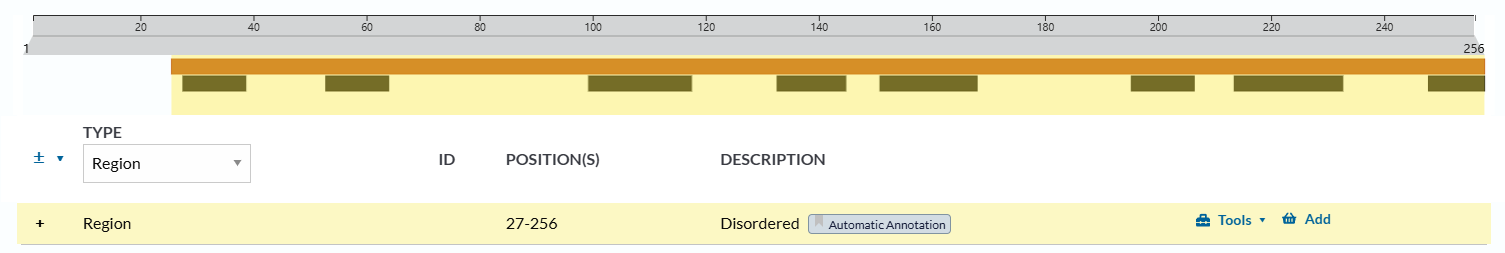


**Supplemental Figure S2** Showing features for region of OsFON879 with UniProt feature annotations. Highlighted regions: Disordered segments (orange) (<https://www.uniprot.org/uniprotkb/Q10PL1/entry>).


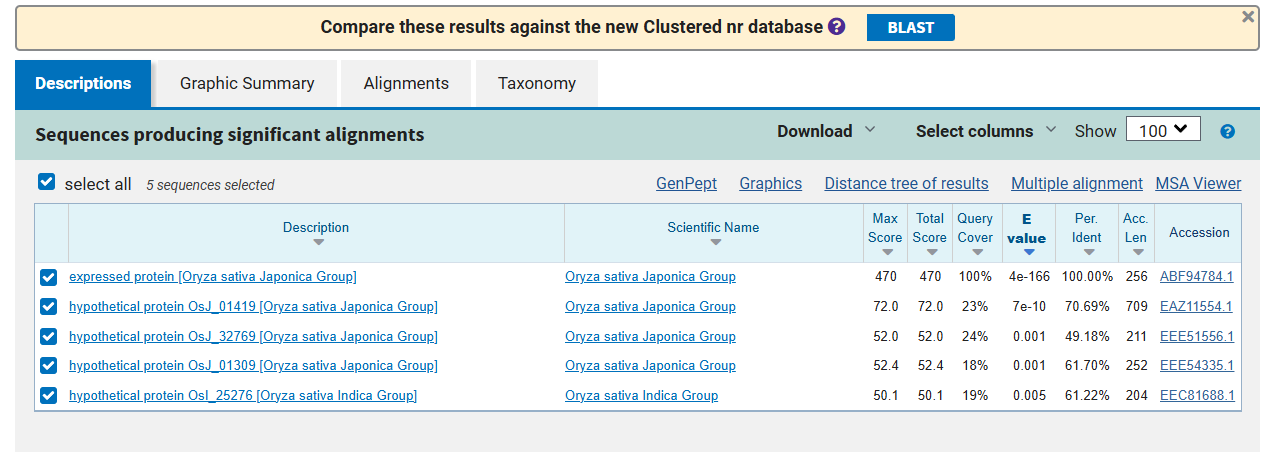


**Supplemental Figure S3** Sequences producing significant alignments with NCBI BLAST (https://blast.ncbi.nlm.nih.gov/Blast.cgi).

**Supplemental Table**

| **Supplemental Table S1. Target sequences used for CRISPR/Cas9-mediated gene knockout** | |
| --- | --- |
| Gene | Target sequence with PAM |
| osfon879-1# | GCCCCGGTCGTCGCCTCGTC CGG |
| osfon879-2# | AGAAGCACCGCGCCTGCGCT CGG |
|  |  |
| **Supplemental Table S2. Primer pairs used in this study** | |
| **Primers for RT-qPCR** | |
| OsFON879RTF | TTGTGGTCTCACGGCTCTAC |
| OsFON879RTR | TTCGGTTTTTCTACAAGCTCGC |
| OsRRM1RTF | GCAGCCGTTCAAGGTATAGGA |
| OsRRM1RTR | CTCGATCTGTCACACGAGCA |
|  |  |
| **Primers for CRISPR-Cas9 mutant and complementation lines genotyping** | |
| GP879F | TGGGCGGATCTACCATAGGA |
| GP879R | CCCCTAAACAACCTGGGACC |
| GPKanF | TGTCATACCACTTGTCCGCC |
| GPKanR | ATCGAGCTGTATGCGGAGTG |
|  |  |
| **Primers for in situ hybridization** | |
| OsFON879-insituSP6F | ATTTAGGTGACACTATAGAATAAAGCACCGCGCCTGCGCTCG |
| OsFON879-insituT7R | AATTAATACGACTCACTATAGGGGGATCTGGCGACGACGACGA |
| OsMADS1-insituSP6F | ATTTAGGTGACACTATAGAATAACCTTGGAGAGGTACCGCAG |
| OsMADS1-insituT7R | AATTAATACGACTCACTATAGGGGGGCCCAAATCCTCACCAAG |
| OsRRM1-insituSP6F | ATTTAGGTGACACTATAGAATAGCAGCCGTTCAAGGTATAGG |
| OsRRM1-insituT7R | AATTAATACGACTCACTATAGGGACACGACCTTCCAACACTGA |
|  |  |
| **Primers for plasmid construction** | |
| pCA12879F | GGGTACCTTGCATATCAAGTGGTGAAGC |
| pCA12879R | CTCTAGAACCTGGGACCCACTGTCAGTC |
| OsFON879-pEGAD-F | GCTGCGGCACCGGCCGAATTCATGACCTTAGCTTGTGG |
| OsFON879-pEGAD-R | CAGTTATCTAGGATCCAAGCTTGTCTCTTCCCATCCCTCTC |
| myc-OsRRM1-F | TCTAGAATGTCGTACTCCAGAGGAT |
| myc-OsRRM1-R | CTCGTTCTGAGCGCTCCCTGGA |
| Enn-OsFON879 | GAGGACTCCGGACTCAGATCTATGACCTTAGCTTGTGGTC |
| Enn-OsFON879 | ATTTTTGCGGACTCTAGATCAGTCAGTCTCTTCCCATCCC |
| Ecc-OsRRM1 | TTCACCATTTACGAACGATAGCATGTCGTACTCCAGAGGAT |
| Ecc-OsRRM1 | GGTCGGCGAGCTGCACGCTGCCTTCTGAGCGCTCCCTGGA |
