# Supplementary material for: OsFON879 , an orphan gene, regulates floral organ homeostasis in rice
Source: Plant Biotechnol J. 2025 May 3;23(7):2888–90. doi: 10.1111/pbi.70121 (PMC12205862; doi:10.1111/pbi.70121)
Supplement: Supplementary file 2 — Data S2. Materials and Methods. [file PBI-23-2888-s002.docx]

**Materials and Methods**

**Plant Materials and Growth Conditions**

The rice (*Oryza sativa*) *cv.* Dongjin was employed for both transgenic studies and as a wild-type control. Seedlings were grown in soil under natural conditions in Beijing’s paddy fields during the growing season or in a climate-controlled chamber (28°C ± 2°C, 11 h light/13 h dark cycle) during winter.

**Generation of CRISPR-Cas9 Mutants and** **Complementation Lines**

To generate *OsFON879* knockout lines, a 23-bp target sequence adjacent to the NGG protospacer adjacent motif (PAM) was designed using CRISPR-P 2.0 (Liu et al., 2017) and cloned into the binary vector pCBSG032 using Golden Gate assembly (Tian et al., 2022). The construct was verified by Sanger sequencing (Supplementary Table S1). The recombinant plasmid was introduced into Agrobacterium tumefaciens strain EHA105 and transformed into rice (*Oryza sativa*) calli following established protocols (Nishimura et al., 2006). Regenerated T0 plants were genotyped by PCR amplification of the target region using primers GP879-F/R (Supplementary Table S2). The PCR products were sequenced using Sanger sequencing to confirm the mutations.

For complementation line construction, a 2,723-bp genomic fragment containing the OsFON879 promoter (1.97 kb upstream) and coding sequence was amplified from Nipponbare genomic DNA using primers pCA12879-F/R (Supplementary Table S2). The fragment was cloned into the KpnI/XbaI sites of the binary vector pCAMBIA2300 and transformed into *osfon879* mutants. Transgenic plants were validated by PCR and sequencing with primers of GPKan-F/R (Supplementary Figure S2).

**RT-qPCR analysis**

Total RNA was isolated from rice tissues (root, leaf, and panicle) using the E.Z.N.A.® Plant RNA Kit (Omega Bio-tek, R6827) according to the manufacturer’s instructions. RNA quality and quantity were determined using a spectrophotometer (NanoDrop). Total RNA used to synthesize cDNA with a qPCR RT Kit (TOYOBO, #FSQ-101) according to the manufacturer’s protocol. RT-qPCR analyses were performed in an ABI7500 machine using TB Green® Premix Ex Taq™ II (Takara Bio, RR420A). *GAPDH* was used as the internal reference gene. Relative expression levels were calculated using the 2^(-ΔΔCt) method (Livak & Schmittgen, 2001). Specific primers for each target gene are listed in Supplemental Table S2. Each experiment was performed with three biological replicates, and each sample was analyzed in three technical replicates.

**Histological Analysis and *In Situ* Hybridization**

Samples of panicles and spikelets at various developmental stages were initially fixed in a FAA solution (comprising 50% ethanol, 10% formaldehyde, and 5% acetic acid, v/v) at 4°C overnight, followed by a series of ethanol dehydration steps. The samples were then embedded in paraffin (Leica, Wetzlar, Germany) for subsequent sectioning.

*In situ* hybridization was carried out according to established methods, with modifications as needed (Wang et al., 2018). DNA sequences specific to the *OsFON879* gene were amplified using specific primers and utilized to synthesize digoxigenin-labeled RNA probes in vitro with the Digoxigenin RNA Labeling Kit (Roche, catalog number 11175025910). Both sense and antisense probes were produced using SP6 and T7 RNA polymerases, with primer sequences provided in Supplementary Table S1. The prepared slides were examined using an Imager.D2 microscope and documented with a ZEISS AxioCam ICC5 digital camera.

**Localization of OsFON879 Protein**

The open reading frame (ORF) of *OsFON879*, excluding the stop codon, was synthesized and inserted into the pEGAD vector to generate a recombinant construct expressing the *OsFON879-GFP* fusion protein. This construct, along with the control pEGAD-GFP, was introduced into *Nicotiana benthamiana* via *Agrobacterium*-mediated transformation. Transgenic plants were grown under controlled environmental conditions (24°C±2°C with a 12-hour light/dark cycle) and analyzed using an Olympus FV3000 confocal microscope for fluorescence signal detection and imaging.

**Yeast Two-Hybrid Assay (Y2H)**

Y2H assays were performed using the Matchmaker GAL4 two-hybrid system as described in the Yeast Transformation System2 user manual (Clontech, Glasgow, UK). The full-length *OsFON879* coding sequence was cloned into pGBKT7, and while the full-length sequences of *OsRRM1* were inserted into pGADT7. The resulting plasmids were co-transformed into the AH109 yeast strain and incubated on selective medium lacking leucine and tryptophan for an initial period of 3 days at 30°C. Subsequently, six independent yeast colonies were selected and further incubated on medium omitting leucine, tryptophan, histidine, and adenine, supplemented with 3-amino-1,2,4 triazole, for an additional 3 days at 30°C to validate potential protein interactions.

**Bimolecular Fluorescence Complementation (BiFC) assay**

For the BiFC assay, the coding regions of *OsFON879* and *OsRRM1* were cloned into the pNC-BiFC-Enn-nEYFP and pNC-BiFC-Ecc-cEYFP vectors, respectively. The *OsFON879* was fused with the N-terminal fragment of *EYFP* (*nEYFP*), and the *OsRRM1* was fused with the C-terminal fragment of *EYFP* (*cEYFP*) (Yan et al., 2020). The plasmids were co-transformed into tobacco leaf epidermal cells using injection method. After 48-72 hours of co-culture, the fluorescence signals were observed using an Olympus FV3000 confocal microscope for fluorescence detection and imaging. The primers used for cloning are listed in Table S2.

**Co-Immunoprecipitation (Co-IP) Assay**

*OsFON879* coding sequence (CDS) was cloned into pEGAD (C-terminal GFP tag) as described above. *OsRRM1* CDS was cloned into pBA-myc (C-terminal myc tag) using KpnI and XbaI sites. The constructed pEGAD-OsFON879 and pBA-myc-OsRRM1 vectors were then transformed into 7-day-old rice (*Oryza sativa*) root protoplasts using PEG-mediated transfection (40% PEG 4000, 0.2 M mannitol, 100 mM CaCl_2_). Transfected protoplasts were incubated in W5 solution (0.5 M mannitol, 4 mM MES, pH 5.7) for 16 h at 25°C in the dark. The protoplasts were harvested, and total proteins were extracted using a protein extraction buffer (100 mM HEPES (pH 8.0), 5 mM EGTA, 5 mM EDTA, 10 mM NaF, 50 mM β-glycerophosphate, 10 mM Na_3_VO_4_, 5% glycerol, 2% Triton X-100, 1 mM PMSF, 1 mM DTT, 1× protease inhibitor cocktail (Roche, 4693159001)) at 4°C. The extracted proteins were used for the Co-IP assay following standard procedures to detect the potential interaction between OsFON879 and OsRRM1 according to the previously described method (Zhang et al., 2018a). 20 µL of agarose-conjugated anti-GFP beads (MBL, D153-10) were added to the IP protein crude extract. The mixtures were gently agitated using a silent mixer at 4°C for overnight. After incubation, the samples were separated on 10% SDS-PAGE gels and transfered to PVDF membranes (Millipore, IPFL00010) for Western Blotting. anti-C-Myc tag Mouse mAb Conjugated HRP antibody (1:3,000, CMCTAG, AT0528), Anti-GFP rabbit polyclonal antibodies (1:3,000, BBI, D110008-0100) were used to detect myc-tagged, and GFP-tagged proteins, seperately. Secondary antibody is HRP-conjugated anti-mouse IgG (1:5,000, ABclonal, AS014). Signals were visualized using SuperSignal™ West Pico PLUS Chemiluminescent Substrate (Thermo Fisher, 34580) and imaged using a ChemiDog™ Imaging System (Tanon 5200T).

**Accession Numbers**

Sequence data from this article can be found in the ricedata libraries under accession numbers *OsFON879* (*LOC_Os03g12879*), *OsRRM1* (*LOC_Os03g15890*) and *OsMADS1* (*LOC_Os03g11614*).

**Supplmental Materials and Methods Reference**

Liu, X., Wu, S., Xu, J., Sui, C., & Wei, J. (2017). CRISPR-P 2.0: An improved CRISPR/Cas9 tool for genome editing in plants. Molecular Plant, 10(3), 530–532. [DOI: 10.1016/j.molp.2016.12.002](https://doi.org/10.1016/j.molp.2016.12.002)

Livak, K. J., & Schmittgen, T. D. (2001). Analysis of relative gene expression data using real - time quantitative PCR and the 2(−Delta Delta C(T)) Method. Methods, 25(4), 402–408. DOI: 10.1006/meth.2001.1262

Wang, D. H., Song, W., Wei, S. W., Zheng, Y. F., Chen, Z. S., Han, J. D., Zhang, H. T., Luo, J. C., Qin, Y. M., Xu, Z. H., & Bai, S. N. (2018). Characterization of the Ubiquitin C - Terminal Hydrolase and Ubiquitin - Specific Protease Families in Rice (Oryza sativa). Frontiers in Plant Science, 9, 1636. DOI: 10.3389/fpls.2018.01636

Yan, P., Zeng, Y., Shen, W., Tuo, D., Li, X., & Zhou, P. (2020). Nimble Cloning: A simple, versatile, and efficient system for standardized molecular cloning. Frontiers in Bioengineering and Biotechnology, 7, 460. DOI: 10.3389/fbioe.2019.00460

Zhang, M., Chiang, Y. - H., Toruño, T. Y., Lee, D., Ma, M., Liang, X., Lal, N. K., Lemos, M., Lu, Y. - J., & Ma, S. (2018). The MAP4 kinase SIK1 ensures robust extracellular ROS burst and antibacterial immunity in plants. Cell Host & Microbe, 24(3), 379–391.e375. DOI: 10.1016/j.chom.2018.08.007
